# Trichothiodystrophy due to *ERCC2* Variants: Uncommon Contributor to Progressive Hypomyelinating Leukodystrophy

**DOI:** 10.1002/mgg3.70067

**Published:** 2025-02-20

**Authors:** Ali Reza Tavasoli, Arastoo Kaki, Maedeh Ganji, Seyyed Mohammad Kahani, Foozhan Radmehr, Pouria Mohammadi, Morteza Heidari, Mahmoud Reza Ashrafi, Kara S. Lewis

**Affiliations:** ^1^ Neurology Division Barrow Neurological Institute, Phoenix Children's Phoenix Arizona USA; ^2^ Myelin Disorders Clinic, Children's Medical Center, Pediatric Center of Excellence Tehran University of Medical Sciences Tehran Iran; ^3^ Department of Medical Genetics, School of Medicine Hamadan University of Medical Sciences Hamadan Iran; ^4^ Research Center for Molecular Medicine, Institute of Cancer, Avicenna Health Research Institute Hamadan University of Medical Sciences Hamadan Iran; ^5^ DeNA Genetics Laboratory Tehran Iran; ^6^ Department of Medical Genetics Tarbiat Modares University Tehran Iran; ^7^ Department of Molecular Medicine National Institute of Genetic Engineering and Biotechnology Tehran Iran

**Keywords:** *ERCC2*, hypomyelination, leukodystrophy, trichothiodystrophy

## Abstract

**Background:**

Trichothiodystrophy (TTD) is caused by homozygous or compound heterozygous variants in genes associated with DNA repair. The *ERCC2* gene encoded a protein, XPD, that is a subunit of the general transcription factor TFIIH and important in both DNA repair and transcription. Disease‐causing variants in *ERCC2* can partially inactivate these activities, giving rise to symptoms seen in TTD, Cockayne syndrome (CS) and xeroderma pigmentosa (XP). Although generalized cerebral white matter abnormalities is reported in TTD, myelination disorders specifically linked to *ERCC2* gene variants are exceptionally uncommon. Here, we introduce a thorough investigation of a patient exhibiting classic TTD symptoms alongside progressive cerebral hypomyelination with *ERCC2* variants.

**Methods:**

In a non‐consanguineous family, we conducted Autism/ID gene Panel on a 5‐year‐old affected child who presented with microcephaly, failure to thrive, developmental delay, and progressive hypomyelination on three serial brain imaging over 5‐years follow‐up. Our investigation aimed to elucidate the genetic underpinnings of the observed phenotype. We also conducted a comprehensive review of the genetic profiles of all documented *ERCC2*‐related patients exhibiting myelination disorders.

**Results:**

Autism/ID gene Panel identified a compound heterozygous variant in *ERCC2* gene causing TTD. Clinical and paraclinical findings enabled differentiation of TTD from Cockayne syndrome and XP. Segregation analysis revealed that, the variation in the paternal allele was a splice junction loss (c.2190 + 1delG), and the other alteration in the maternal allele was a pathogenic variant (c.1479 + 2dupT). It has been noted that these variants were reported in previous studies in homozygous or compound heterozygous form in patients with TTD, but none of them exhibited hypomyelinating leukodystrophy.

**Conclusion:**

The identification of hypomyelination in TTD due to *ERCC2* sheds a light on the molecular diagnosis and contributing to the limited literature on *ERCC2* variants and associated hypomyelinating leukodystrophy in patients with TTD.

## Graphical abstract

This study reports a novel case of trichothiodystrophy (TTD) linked to compound heterozygous ERCC2 variants, presenting with progressive cerebral hypomyelination. The findings highlight a rare association between ERCC2 mutations and hypomyelinating leukodystrophy, expanding the current understanding of TTD‐related neurodevelopmental disorders.

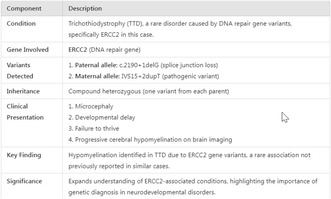

## 1 Introduction

Trichothiodystrophy (TTD) (OMIM #601675) is a rare genetic disorder which is characterized by brittle hair due to a deficiency in sulfur and cysteine, leading to a condition known as Trichoschisis (Itin, Sarasin, and Pittelkow 2001). When observed under polarized light microscopy, the hair displays alternating dark and light bands, referred to as ‘tiger tail banding’ (Liang et al. 2005). The main clinical variants of TTD include photosensitive TTD, non‐photosensitive TTD, and a combination of both, known as mixed TTD. Fifty percent of individuals affected by TTD experience the photosensitive type, which makes them highly reactive to ultraviolet (UV) rays. Even brief exposure to sunlight can result in intense sunburn for these individuals (Lambert, Gagna, and Lambert 2010). The photosensitive variant of TTD is linked to disease‐causing variants in three specific genes: *XPB* (*ERCC3*) (OMIM*133510), *XPD* (*ERCC2*) (OMIM*126340), and *GTF2H5* (*p8/TTDA*) (OMIM*608780). TTD is a distinct condition from Xeroderma Pigmentosum (XP) caused by mutation in eight XP‐associated genes: XPA, XPD (ERCC2), XPB (ERCC3), XPF (ERCC4), XPG (ERCC5), XPC, XPE (DDB2), and a variant form related to the *POLH* gene. While most individuals with TTD are sensitive to sunlight, they do not have a higher risk of developing cancer. In addition, the key features of TTD include brittle hair that lacks sulfur, neurological developmental delays and, physical growth impairment, skin scaling (ichthyosis), and unique facial characteristics, which are absent in XP (Chatterjee and Walker 2017).

The three genes responsible for the photosensitive variant of TTD are involved in encoding different subunits of the basal transcription factor II H (pTFIIH) complex, which is composed of 10 subunits. These genes play a crucial role in the process of nucleotide excision repair and basal transcription, and repairing DNA damage, which can be caused by UV rays (Hashimoto and Egly 2009).

Overall, TTD presents with a variety of clinical symptoms, including cutaneous, neurologic, and growth abnormalities. Frequently observed clinical characteristics are intellectual/developmental disabilities, microcephaly, ichthyosis, short stature, unique facial characteristics, and susceptibility to infections. Less commonly reported symptoms are reduced fertility, multisystem abnormalities, and eye‐related abnormalities (Stefanini et al. 2010). It is also important to distinguish TTD from Cockayne syndrome. Both conditions share several clinical features, such as skin photosensitivity, short stature, neurological abnormalities, and microcephaly (Farmaki et al. 2017). However, Cockayne syndrome is specifically linked to variants in the *ERCC6* (OMIM*609413) and *ERCC8* (OMIM*609412) genes. Indicators of Cockayne syndrome include the presence of cataracts and/or retinopathy, bone abnormalities, and white matter signal changes in brain magnetic resonance imaging (MRI) indicative of leukodystrophy. Brittle hair, which is a characteristic feature suggestive of TTD, is absent in Cockayne syndrome (Morice‐Picard et al. 2009).

Herein, we introduced a thorough investigation of a 5‐year‐old male patient exhibiting classic TTD symptoms alongside progressive cerebral hypomyelination arising from *ERCC2* disease‐causing variants. To evaluate the frequency of the cerebral myelination disorders in TTD, we conducted a comprehensive review of the literature to identify all published case reports involving TTD patients with hypo/demyelination or white matter signal changes caused by *ERCC2* gene variant (Usuda et al. 2011; Ji et al. 2018). To date, it has only been observed in four reported cases. Therefore, this study underscores the significance of disorders related to hypomyelinating leukodystrophy as a clinical phenotype of TTD.

## 2 Materials and Methods

### 2.1 Ethical Compliance

Legal guardians provided written informed consent for participation in this study and the publication of photographs. The study adhered to the Declaration of Helsinki guidelines and received approval from the Phoenix Children's Institutional Review Board under the code IRB‐ 24‐065.

### 2.2 Subjects and Clinical Evaluation

The initial genetic counseling was provided to a family with an 8‐month‐old child displaying developmental delay, microcephaly, feeding problems, failure to thrive, and an undescended left testis in order to establish a molecular diagnosis. Subsequently, a patient chart review spanning over 4.5 years from the initial visit was conducted. This comprehensive review included multispecialty initial and follow‐up visits to extract the required genomic, clinical, and imaging data of the patient. At the time of study, the patient was a 5‐year‐old male patient and had undergone various assessments and treatments since first evaluation, forming the basis of our case presentation.

### 2.3 DNA Methylation and Methylation‐Specific PCR (Ms‐PCR)

Extracted DNA from the patient treated with Sodium bisulfite for methylation‐specific polymerase chain reaction (ms‐PCR) and digest with the restriction endonuclease Eae1 and lpnp1foe RD‐PCR analysis.

### 2.4 Sequence Analysis for Epilepsy Panel

The genomic DNA extracted from the affected sample underwent a process of targeted enrichment using a hybridization‐based protocol. Subsequently, it was sequenced using Illumina technology. Unless otherwise specified, all the regions of interest including 180 genes were sequenced with a depth of 50× or greater, and additional analysis was performed. The sequencing reads were then aligned to a reference sequence (specifically, GRCh37). In the context of a clinically relevant transcript, any sequence changes were identified and interpreted. The focus of this enrichment and analysis lies on the coding sequence of the indicated transcripts, along with 10 base pairs of flanking intronic regions.

### 2.5 Autism/ID Gene Panel and Co‐Segregation Analysis

The genomic DNA extracted from the provided sample was comprehensively enriched to cover all coding regions and the splice sites junctions for the most of genes within the human genome. This was achieved through a specialized capture system on Illumina platform, exclusively developed by GeneDx, which is utilized for advanced next‐generation sequencing that includes copy number variation detection (NGS‐CNV). Paired‐end sequence reads were assembled and aligned to human genome build GRCh37/ hg19. Data filtering and analysis to identify sequence variants were performed using a custom‐developed analysis tool. To study the segregation analysis, Sanger sequencing was used to sequence the detected disease‐ causing variant and it's flanking genomic regions.

### 2.6 Literature Review

A thorough investigation was conducted across multiple databases including PubMed and Web of Science up to March 2024. We utilized specific keywords such as “*ERCC2* [title/abstract] mutation”, “XPD [title/abstract] mutation”, “Trichothiodystrophy [title/abstract] mutation”, “TDT [title/abstract] mutation”, and “myelination [title/abstract] mutations”. Our goal was to identify all documented patients with *ERCC2* (*XPD*) variants. We carefully extracted data from original studies, covering demographics, clinical presentations, family histories, myelination phenotypic manifestations, imaging findings, genotype/zygosity, and the pathogenicity of detected variants in affected individuals (Table 1).

**TABLE 1:** Clinical/genetic findings in trichothiodystrophy (TTD) patients with myelination disorders caused by *ERCC2* variants.

Patients ID		Patient 1	Patient 2		Patient 3	Patient 4		Present study	
Age, sex		10 years/male	4 months/male		5 months/male	NA		8 months/male	
Origin		Caucasian	Japanese		Greek	Chinese		American	
Consanguinity		No	No		No	NA		No	
Variant	cDNA	c.2164C>T	Paternal	c.2164C>T	c.335G>A	Paternal	c.1808_1809del	Paternal	c.2190 + 1delG
		c.1133G>A	Maternal	NA		Maternal	c.2164C>T	Maternal	c.1479 + 2dupT
	Protein	p.R722W	Paternal	R722W	p.R112H	Paternal	p. K603Sfs*45	Paternal	p.E731Rfs*14
		p.R378H	Maternal	p.Ser23del		Maternal	p.R722W	Maternal	—
	Zygosity	Compound heterozygous	Compound heterozygous		Homo	Compound heterozygous		Compound heterozygous	
Clinical features		Photosensitivity, ichthyosis without the increased freckle‐like pigmentation of XP, short brittle hair with tiger tail pattern, short stature	Low birth way, microcephaly, ichthyosis, erythroderma, thin, spare, and brittle hair, hair shaft abnormalities (trichoschisis, trichorrhexis nodosa‐like fracture and ribboning), red skin rash on his face, solar dermatitis with delayed erythema after short sun exposure, hypoplastic nails		Hypertonia, microcephaly, microphthalmia, hypertelorism, sparse eyebrows, large and protruding ears, high arched palate, thin nails thin, spare, and brittle hair	Microcephaly, ichthyosis, Brittle hair, spastic quadriplegia		Hypotonia, failure to thrive, short stature, microcephaly, congenital cataract	
Neurologic findings		Sensorineural hearing loss, developmental delay	Developmental delay		Growth retardation	Mental retardation		Global developmental delay, speech delay, conductive hearing loss	
MRI features		Reduced myelination of the brain	Dysmyelination		Hypomyelination	Hypomyelination		Hypomyelination	
Other phenotypes		Multiple sinopulmonary infections	Chronic neutropenia at 14 months of age, recurrent infections		Neutropenia, hypogammaglobulinemia, recurrent infections	—		Hypogammaglobulinemia, recurrent ear infections	
Reference		Tolmie et al. (1994)	Usuda et al. (2011)		Farmaki et al. (2017)	Ji et al. (2018)		Present study	

## 3 Results

### 3.1 Clinical Findings

The patient was born at 37 weeks gestation via normal vaginal delivery as the second child of a non‐consanguineous parents. Mother's and father's ethnicity were European and European/Native American, respectively, who referred to the Genetics and Metabolism Clinic of Phoenix Children's Hospital at the age of 8 months. His birth weight was 2360 g (low birth weight), length was 45.72 cm, and head circumference was 30.48 cm (*z* score: −2.83, less than 3rd percentile). His older sister was normal with no other problems in the family.

**FIGURE 1:**
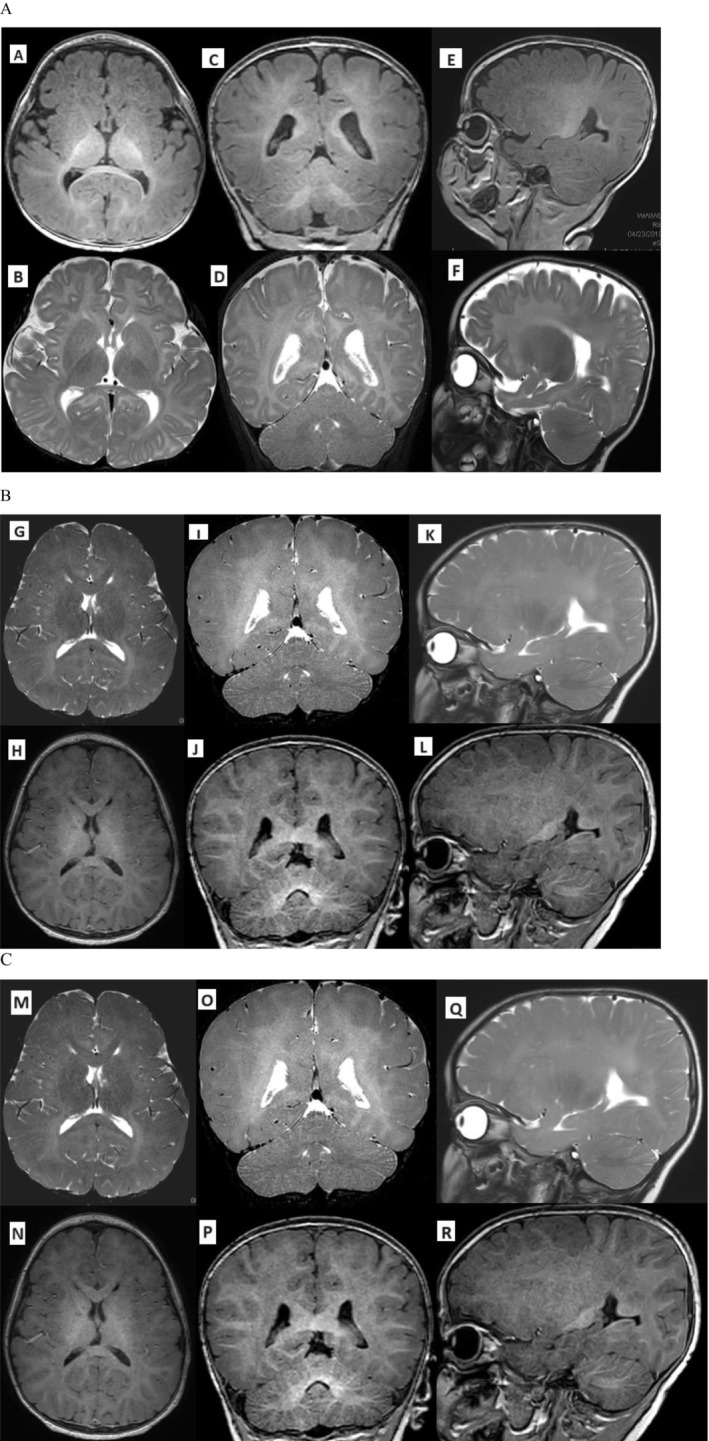
Progressive hypomyelination over serial brain imaging spanning over 4.5 years. (A) Brain MRI (8 months) demonstrated unremarkable imaging findings on axial, coronal and sagittal T1‐weighted (A, C, E) and correspondent T2‐weighted sequences (B, D, F). (B) Brain MRI (at 22 months) revealed deep and bilateral subcortical frontal, parietal, and occipital hyperintense signals on T2‐ weighted images and to lesser extent deep periventricular white matter hypomyelination. The axial, coronal and sagittal T2‐ weighted spaces (G, I, K) and correspondent T1‐weighted images (H, J, L) were normal. (C) Brain MRI (4 years, 2 months) showed diffuse abnormal increased signals on T2‐weighted images in the supratentorial white matter and poor gray‐white—differentiation on T1‐weighted images compatible with hypomyelination. There appears to be further interval loss of normal myelination compared to the prior exams at ages 22 and 8 months old including regions of subcortical white matter abnormal signal currently, previously demonstrating a more normal signal. Overall cerebral volume appears normal T2‐weighted images (M, O, Q) and corresponding T1‐weighted images (N, P, R).

During the first 4 weeks of his life, he was very sleepy and hard to wake up and showed sleep disturbance. He moved all night long with snoring. At 8 months of life, during the initial neurology clinic visit, he was diagnosed with hypotonia, microcephaly, global developmental delay, poor head control, swallowing problem and feeding difficulties (G‐tube in place), failure to thrive from the beginning, short stature, left undescended testis, penile adhesions, and abnormal involuntary movements. In addition, atopic dermatitis with a dry, cracked, and itchy skin lesions was detected on his face and trunk examination. His gross hair exam was normal. The presence of a few clinical findings like small hands and feet, bitemporal narrowing, and almond shaped eyes was suggesting a diagnosis of Prader‐Willi syndrome. Therefore, the methylation testing was tested during the initial genetic consultation to make a molecular diagnosis. Other suggested disorders in patient were Angelman and Rett syndrome as well as epilepsy syndromes presenting with an unusual presentation, although the family did not report any seizure or seizure‐like phenomena during the initial consultation. The Invitae sequencing panel consist of 180 genes for these syndromes was normal. He has also had normal methylation analysis for Prader‐Willi syndrome. An initial brain MRI without contrast at the age of 8 months old demonstrated unremarkable imaging findings on axial, coronal and sagittal T1‐Weighted (A, C, E) and correspondent T2‐Weighted sequences (B, D, F) (Figure 1A).

At 1 year and 8 months of his life, he remained developmentally delayed with no regression, unable to stand and walk in addition to speech delay. However, he experienced recurrent ear infection with normal immune deficiency test results. He was treated with a short‐term immunoglobulin therapy which resulted in significant improvement. He was also re‐evaluated for seizures identifying and his electroencephalogram (EEG) was normal. He had difficulty with feeding; hence he was entirely dependent on continuous tube feeding. During the follow‐up neurology clinic visit at 22 months old, his second brain MRI results revealed deep and bilateral subcortical frontal, parietal, and occipital hyperintense white matter signals on T2‐Weighted images and to lesser extent deep periventricular white matter signal changes due to abnormal for his age white matter tracts (hypomyelination). Similarly, the T1‐weighted signals were not as hyperintense as one would expect for a patient at this age. Brain volume appeared normal. The basal ganglia, cerebellum and vermis were normal. The size of the ventricles were normal. A right choroidal fissure cyst measures 7 mm also detected. The extra‐axial spaces were normal (Figure 1B), axial, coronal and sagittal T2‐Weighted (G, I, K) and corresponding T1‐weighted images (H, J, L). In addition, regarding the sleep problems, an overnight polysomnogram was conducted that showed mild obstructive sleep apnea becoming more severe in the REM sleep state. In terms of developmental milestones, he was behind but was slowly making progress at his own pace that is, pulling to stand and holding onto the furniture. He had no regression. There had been no definite staring spells, loss of consciousness, or concerns about seizures. He had been undergoing physical therapy. He was able to imitate car sounds but no other sounds. He had a social smile and interacted with adults and kids; however, he occasionally had tantrums.

At the age of 2 years, an ophthalmology consultation revealed a congenital cataract, which was not visually significant, along with suspected bilateral amblyopia. Glasses were prescribed. In addition, audiology evaluation detected a mild bilateral conductive hearing loss resulting in bilateral ear tube placement. At age 4 year and 2 months old, a third brain MRI without contrast showed diffuse abnormal increased signals on T2‐weighted images in the supratentorial white matter and poor gray‐white differentiation on T1‐weighted images compatible with hypomyelination. There appeared to be further interval loss of normal myelination compared to the prior exams at ages 22 and 8 months old including regions of subcortical white matter abnormal signal currently, previously demonstrating a more normal signal. Overall cerebral volume appeared normal (Figure 1C), axial, coronal and sagittal T2‐weighted images (M, O, Q) and corresponding T1‐weighted images (N, P, R). Developmentally, he was getting close to walking. He was cruising well, took five steps independently, walked well with two hands, and occasionally let go of one. He was receiving therapies both in and out of school, including physical therapy (PT), speech therapy (ST), and occupational therapy (OT). He made noises, could point to things, understood more than some people realized, and recognized individuals.

Additional genetic testing (GeneDx Autism/ID Xpanded Panel) was conducted considering unexplained global developmental delay and progressive hypomyelination over serial brain imaging spanning over 4.5 years. The results showed “two pathogenic variants in the *ERCC2* gene which were biparentally inherited.” This gene is consistent with three different clinical presentations including cerebro‐oculo‐facio‐skeletal syndrome (COFS), xeroderma pigmentosum, and TTD. Based on the clinical and imaging findings, the patient was referred for further evaluations by dermatology, ophthalmology as well as audiology specialists to consider TTD. He underwent a brainstem auditory‐evoked response (BAER) study following bilateral placement of ear tubes, the results of which were most consistent with a bilateral, mild, conductive hearing loss. On further ophthalmology clinic visit, mild stable lamellar congenital cataract, mild bilateral amblyopia as well as myopia were diagnosed. No changes were made in eyeglasses. In dermatology clinic visit, bilateral xerosis cutis, melanocytic nevi of unspecified on the upper and lower limbs, and scalp and neck were detected. Additional findings were stereotypic movements and habit disorder, and esophageal reflux. Taken all these findings together, the patient was diagnosed with TTD as neurological findings, physical growth impairment, ichthyosis, and unique facial characteristics as the main findings of TTD are absent in XP. In addition, progressive microcephaly and hypotonia, micro‐ophthalmia and optic atrophy, and joint contracture as the cardinal findings of COFS were absent in our patient.

### 3.2 Ms‐PCR and RD‐PCR Findings

These tests detect approximately 70% of patients who meet the clinical criteria for Angelman syndrome (AD) and approximately 99% of patients who meet the clinical criteria for Prader‐Willi syndrome. It is important to note that these tests do not differentiate deletions, uniparental disomy, and imprinting variants as the causes of abnormal small nuclear ribonucleoprotein polypeptide N (SNRPN) methylation. Both methods allow detection of parent‐specific methylation of the SNRPN gene in which normal individuals have methylated and unmethylated products observed at equal intensities (methylation index=50). The methylation index of ms‐PCR and RD‐PCR was 0.57 and 0.46 respectively and the results of ms‐PCR and RD‐PCR were consistent with normal biparental inheritance and did not indicate a diagnosis of AD or Prader‐Willi Syndrome (PWS).

### 3.3 Invitae Panel Result

This panel list of genes consists of preliminary evidence genes for epilepsy, Glycine Encephalopathy, *FLNA*, *PTEN*, and *RANBP2* genes as well as Rett and ADs and related disorders. This diagnostic test examines 188 genes for alterations that are linked to the mentioned genetic disorders. This gene‐panel test did not identify any pathogenic variants but includes at least a heterozygous VUS variant (c.1667G>C, p.Arg556Thr) in *CARS2* gene. The *CARS2* gene is associated with autosomal recessive combined oxidative phosphorylation deficiency (OMIM#616672) that is not completely related to the clinical findings of this patient.

### 3.4 Molecular Genetics Diagnosis

Two heterozygous pathogenic variants in the *ERCC2* gene were identified through Autism/ID Gene Panel. Considering compound heterozygosity for these variants, the genetic diagnosis of autosomal recessive ERCC2‐related disorder was possible. Considering the reported clinical findings, TTD is the most likely diagnosis. Segregation analysis by Sanger sequencing revealed that the variation in the paternal allele was a splice junction loss (c.2190 + 1delG), and the other alteration in the maternal allele was a pathogenic variant in *ERCC2* (c.1479 + 2dupT).

### 3.5 Single‐Cell RNA‐Sequencing of ERCC2 in Human and it's Orthologous in Other Species

**FIGURE 2:**
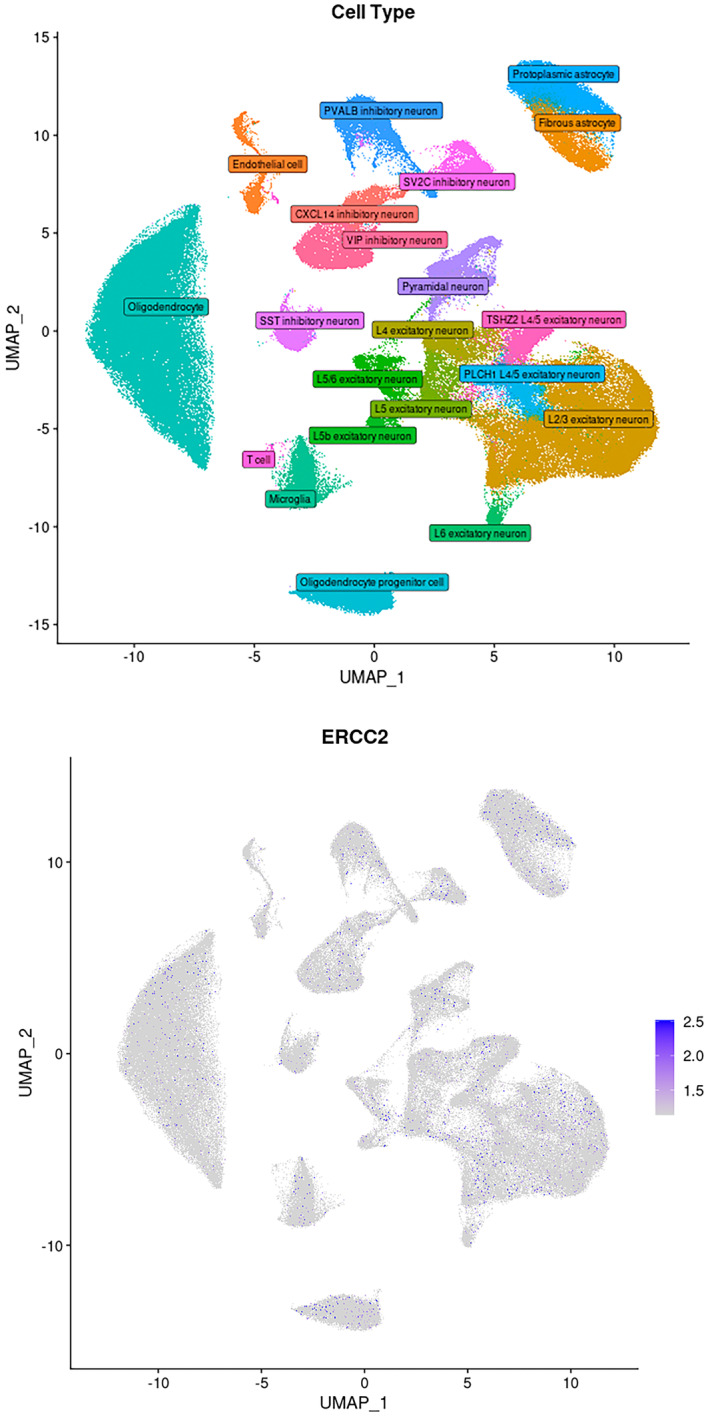
Publicly available single‐cell sequencing database, recruited to investigate the cell type gene expression of ERCC2 in human brain. ERCC2 is widely expressed in a range of neurons, fibroblast and mesenchymal lineage biology. The cell type expression of ERCC2 shows this gene is expressed in most or all regions of the brain suggest that this gene is important for central nerve system development. This statement supported by gene expression pattern of ERCC2 paralogous in other specious as this gene is highly conserved during the evolution.

Publicly available single‐cell RNA sequencing database, database of Deeply Integrated human Single‐Cell Omics, DISCO, was recruited to investigate the cell type gene expression of ERCC2 in human brain. As shown in Figure 2, ERCC2 is widely expressed in most or all regions of the brain. The ERCC2 is expressed in a range of neurons, fibroblast and mesenchymal lineage biology. These data suggesting that ERCC2 could be important for central nerve system development (Human: https://www.immunesinglecell.org/).

**FIGURE 3:**
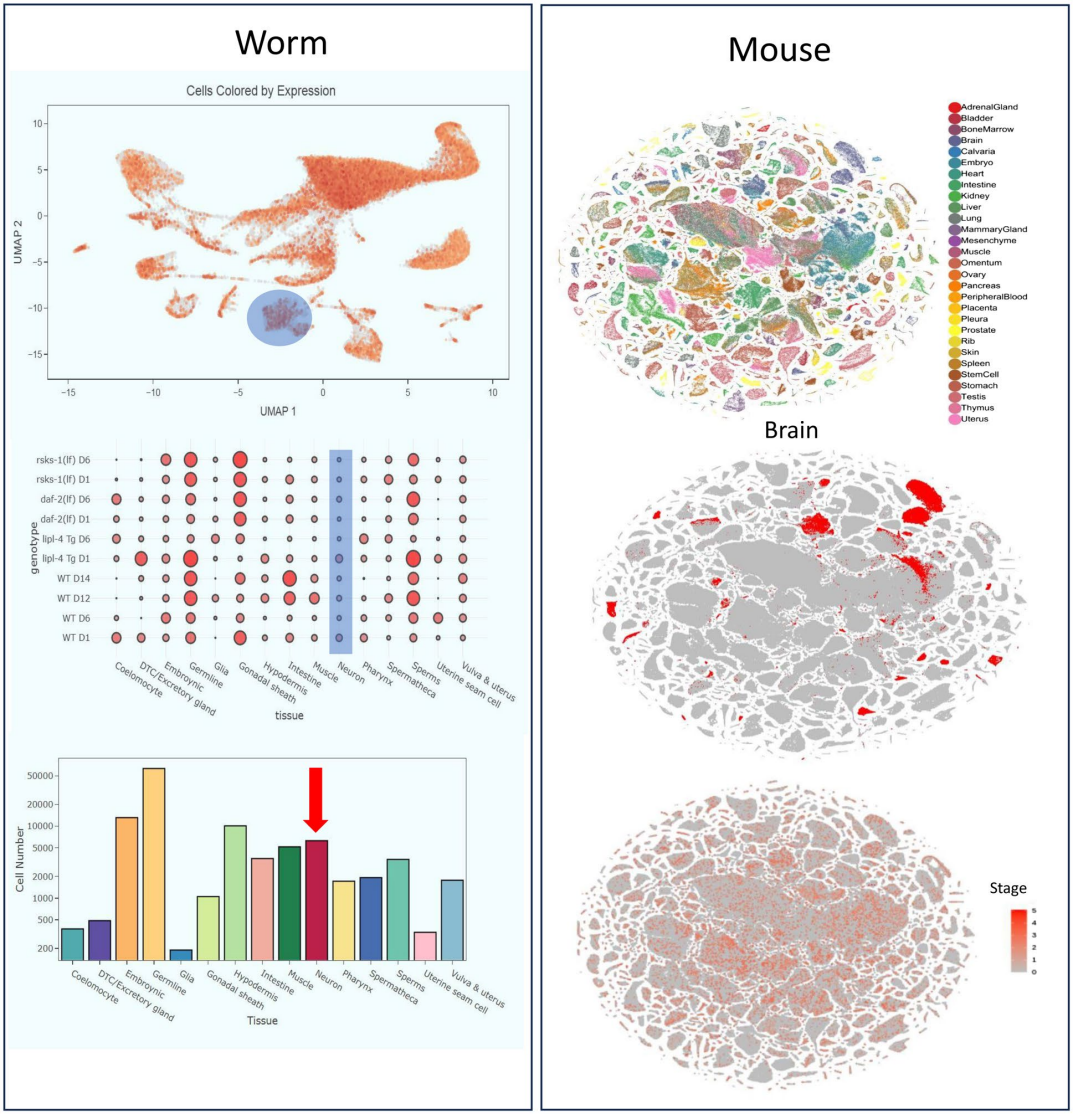
ERCC2 remains conserved during evolution through different organisms. Single cell transcriptome sequencing in C.elegans shows that xpd‐1, ortholog of ERCC2, is widely expressed in Germline and neurons. In addition, Publicly available single‐cell sequencing of mouse shows that Ercc2, ortholog of ERCC2 in mouse, indicates that this gene is broadly expressed through the body, including neuronal cells in the brain.

Single cell transcriptome sequencing in *C. elegans* shows that xpd‐1, orthologue of ERCC2, is widely expressed in neurons of the central nervous system (CNS) (Worm: https://agingc.shinyapps.io/Aging_Atlas/). In addition, publicly available single‐cell sequencing of mouse shows that Ercc2, ortholog of ERCC2 in mouse, indicates that this gene is broadly expressed through the body, including neuronal cells in the brain (Mouse: https://bis.zju.edu.cn/MCA/atlas3.html/) (Figure 3).

## 4 Discussion

Trichothiodystrophy is a rare autosomal recessive disorder caused by variants in at least 10 genes. The photosensitive variant of TTD is primarily caused by variants in one of three genes: *ERCC2* (*XPD*), *ERCC3* (*XPB*), or *GTF2H5* (*p8/TTDA*) (Kleijer et al. 2008; Botta et al. 2009). These genes encode proteins that are part of the general transcription factor 2 H (pTFIIH) complex. This complex plays a crucial role in repairing DNA damage, which can be caused by UV rays. Additionally, the TFIIH complex is significantly involved in gene transcription, which is the first step in protein production (Hashimoto and Egly 2009).

**FIGURE 4:**
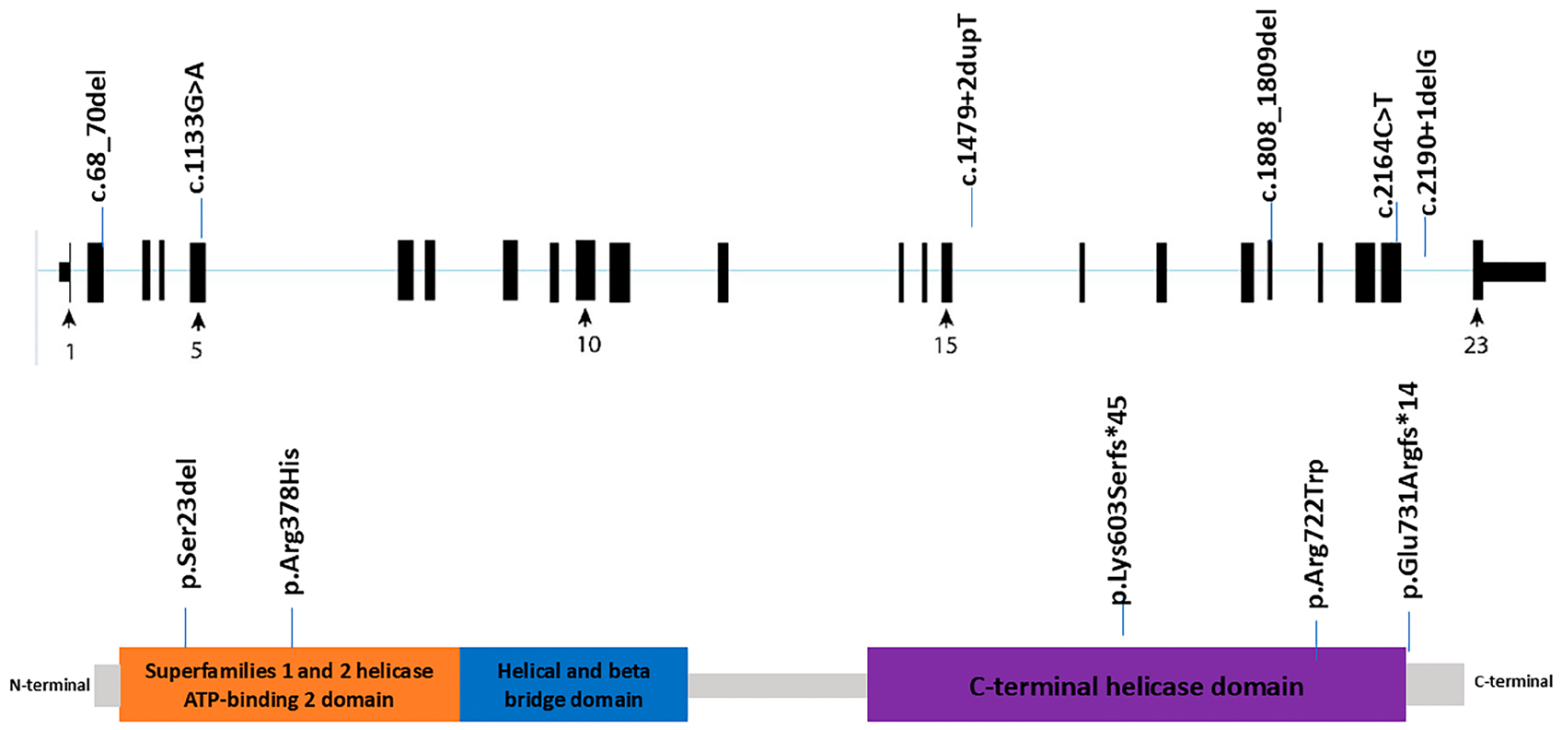
The schematic representation of the *ERCC2* gene and protein domains. The upper part shows the exon structure of the *ERCC2*, while the lower part illustrates the protein domains and their respective positions along the amino acid sequence. Specific domains such as the ATP‐binding type‐2 domain and the helical and beta‐bridge domain are highlighted along with the C‐terminal helicase domain of xeroderma pigmentosum. Variants are marked on both the gene and protein level diagrams: Frameshift and in‐frame variants are shown in red, and missense variants are displayed in purple. This schematic effectively maps variants to specific exons and protein domains, indicating how genetic alterations could potentially influence protein function.

**FIGURE 5:**
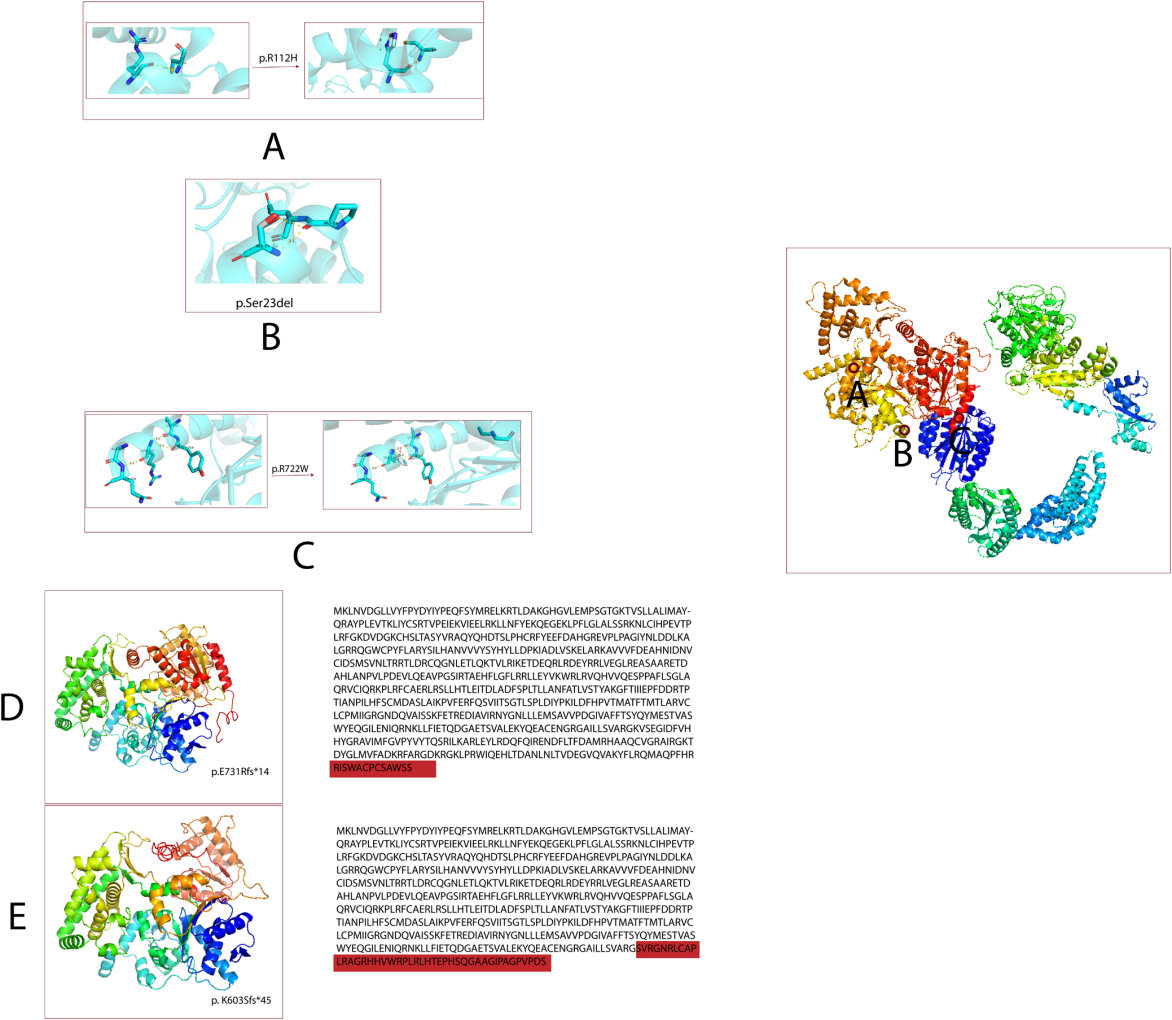
Visualization of amino acid interactions in ERCC2 protein structures using PyMOL, based on the PDB5IVW model from the Protein Data Bank. Each section illustrates specific changes in amino acid residues documented in the literature: Interaction changes of p.R112H before and after variation (A), interaction of Ser23 before deletion (B), and p.R722W before and after variation (C). Additionally, we used the I‐TASSER protein prediction database to model the structural impacts of frameshift variants, including p.E731Rfs14 (E) and p.K603Sfs45 (D). Affected amino acids are highlighted in red within the sequence alignments, illustrating the connection between sequence alterations and structural deviations. This comprehensive representation elucidates the potential functional impacts of these genetic changes on protein architecture and functionality.

The *XPD* gene, also referred to as *ERCC2*, is located on chromosome 19q13.2. It codes for a 5′–3′ helicase, which is a component of the transcription factor TFIIH, consisting of 760 amino acids. This gene consists of 23 exons (De Boer et al. 1998). Disease‐causing variants in the *ERCC2* gene are linked to the TTD, a condition that manifests in various clinical symptoms such as cutaneous, neurological, and growth abnormalities (Figures 4 and 5) (Nakano et al. 2014). The main clinical features of TTD consist of developmental disabilities, microcephaly, ichthyosis, short stature, unique facial characteristics, and susceptibility to infections. Eye‐related abnormalities are less commonly reported symptom (Faghri et al. 2008).

Regarding the variation in the paternal allele (c.2190 + 1delG), it is in a canonical (NM_000400.4 | ENST00000391945) splice‐site and is anticipated to influence mRNA splicing. This could lead to a significantly modified protein due to either exon skipping, shortening, or the inclusion of intronic material. Multiple computational tools predict a substantial impact on normal splicing: four of them predict that the variant eliminates the canonical 5′ splice donor site, while four others predict the creation of a new 5′ donor site (Broughton et al. 1994). However, these predictions are yet to be validated by functional studies. The c.2190 + 1delG has been documented in the literature under various legacy naming conventions such as c.2189delG in Lehmann et al. (TTD1B1 case) and Tolmie et al. (TTD2BR) studies which were reported in homozygous and compound heterozygous, respectively (Lehmann et al. 1988; Tolmie et al. 1994), and c.2190delG in Boyle et al. (2008) study.

In TTD2BR patients, in addition to the “G2268 frameshift”, there was a second variant involving an 18 bp deletion at bases 1540–1557. This deletion leads to the in‐frame loss of amino acids 488–493. This was demonstrated in a study by Broughton et al. in individuals affected by TTD (Broughton et al. 1994). The translational impact has been reported as (p.E731Rfs*14) (Faghri et al. 2008). Based on the ACMG/AMP criteria, the classification of c.2190 + 1delG is likely to be pathogenic due to the compelling evidence from PVS1, supported by other criteria such as PM2, PP3, PS3.To the best of our knowledge, no experimental evidence demonstrating an impact on protein function has been reported.

The c.1479 + 2dupT variant in the maternal allele has been documented in a study involving two unrelated individuals diagnosed with TTD (Broughton et al. 1994). Both individuals were compound heterozygous for this variant, one with a deletion variant on the second allele and one with a missense variant. RT‐PCR studies revealed an 18 bp deletion caused by the c.1479 + 2dupT variant, leading to an in‐frame loss of six amino acids in exon 15. This variant is in the intron region (± 1, 2) of the splice consensus sequence and is predicted to cause altered splicing, leading to an abnormal or absent protein. In summary, while further studies are needed to fully determine its clinical significance, the c.1479 + 2dupT variant is likely pathogenic. The ACMG/AMP Criteria applied include: PM2; PM3; PM4. Functional studies have shown the creation of two mRNA products; one resulting from the loss of exon15 and another due to an 18 bp deletion caused by using a cryptic splice site (Broughton et al. 1994).

In the studies by Lehmann and Tolmie et al., no abnormalities in cerebral white matter were reported in their TTD patients with a variant similar to that of our case. They presented a 6‐year‐old boy (TTD1B1 patient) suffering from brittle hair and Trichorrhexis nodosa. At birth, he exhibited the typical “collodion baby” appearance, with a slightly unusual face and brittle, stubbly hair. He also had nystagmus and remnants of cataracts, which were surgically removed at 3 months old. He displayed dysplastic nails and an ichthyotic skin pattern, but there were no signs of photosensitivity. Throughout infancy, he experienced numerous respiratory infections and failure to thrive. The other patient (TTD2BR), a second‐born twin delivered at full term baby. At birth, she had an unusual hair texture, fragile nails, and dry skin. Her weight and head circumference were 3 standard deviations below the norm by 17 months of age. By 2.5 years, she could sit without support and move by bottom shuffling. A neurological examination revealed signs of pyramidal tract involvement, including symmetrically increased muscle tone, heightened tendon reflexes in all limbs, and extensor plantar responses. No cataracts were detected. A developmental assessment indicated a developmental quotient of 50 (Lehmann et al. 1988; Tolmie et al. 1994).

Boyle et al. reported a six‐year‐old boy with TTD due to the same variant (c.2190delG) of our patient who presented with severe photosensitivity, but no freckle‐like pigmentation was reported. Additional symptoms included short, brittle hair with a ‘tiger tail’ pattern, cataracts, recurrent ear infections and conducting hearing loss, microcephaly, developmental delay, and decreased brain myelination. However, no follow‐up imaging was reported (Boyle et al. 2008). Dysmyelination was diagnosed in another Japanese patient at 18 months of age through brain MRI, revealing a compound heterozygous variant (c.2164C>T; p. Arg722Trp & c. 67_69del; p. Ser23del) different from our patient's variant (Usuda et al. 2011). In a young infant Greek case genetic testing detected a homozygous variant (c.335G>A; p.Arg112His) in exon 5 and, brain MRI showed delayed myelination at 2 months of age, with a follow‐up MRI at 16 months confirming the initial findings of delayed hypomyelination (Farmaki et al. 2017) (Table 1).

TTD is a rare genetic disorder which often causes intellectual disability and developmental delay, and its hallmark symptom is sparse and easily broken hair. Individuals with TTD may exhibit brain abnormalities that can be detected through imaging tests. To assess the most common reported neurological symptoms, we have summarized results of a thorough investigation conducted on 112 patients with TTD. The study found that 86% of the patients showed signs of developmental delay or intellectual impairment. Within this group, 41 patients also had impaired motor milestones or psychomotor retardation. Other abnormal neurological findings included microcephaly in 50% of patients, abnormal gait in 26%, and heightened deep tendon reflexes in 13%. Neuroimaging abnormalities were reported in 23% of the patients, with the most common findings being dysmyelination (14%), cerebellar atrophy (4%), and dilated ventricles (4%) (Faghri et al. 2008). Therefore, the common imaging finding of this complex neurological condition is impaired myelin production which has been described as dysmyelination in a few publications (Broughton et al. 1994; Lehmann et al. 1988; Harreld et al. 2010). To date, myelination impairment has only been reported in four cases of TTD in which *ERCC2* variants are responsible for the disease (Farmaki et al. 2017; Ji et al. 2018; Boyle et al. 2008). However, in none of these reported cases, has persistent and progressive hypomyelination has been reported on serial brain imaging over time after the age of 2.

In addition to the TTD, sulfur‐deficient hair and white matter abnormalities are also observed in Cockayne syndrome. The impaired myelin production seen in TTD has been linked to the impaired transcription of myelin's structural components and molecules with a high sulfur content, such as neurocan and phosphacan, which are crucial for the development of the CNS and brain cortex (Maeda 2015; Meyer‐Puttlitz et al. 1996; Porto et al. 2000). Therefore, hypomyelination could be considered as a better description of white matter abnormality as it affects the initial steps of CNS white matter development.

In the absence of gray matter abnormalities in children, other conditions that could be considered in the differential diagnosis for widespread hypomyelination include Pelizaeus‐Merzbacher disease, 18q‐syndrome, and Salla disease. Although congenital metabolic disorders can cause delayed myelination that resembles the widespread impaired myelin abnormalities seen in TTD (TTD), cortical atrophy and unique clinical and imaging findings usually enable differentiation between TTD and these disorders (Harreld et al. 2010). In practical terms, the main diagnostic criteria for TTD continue to be the clinical and pathological observations, and MRI serves as a useful tool for obtaining supportive. Clinically, compared to the other four cases with myelination disorder, our patient showed a progressive hypomyelination on serial MRIs spanning over 4.5 years in addition to the common symptoms of TTD including psychomotor delay, microcephaly, growth impairment, unique fascial features, congenital cataracts and hearing loss.

In conclusion, TTD, COFS, XP, and Cockayne syndrome are distinct yet overlapping neurocutaneous disorders, each presenting a unique constellation of clinical features rooted in shared molecular pathways of DNA repair and oxidative stress. These disorders can be differentiated based on key features: Xeroderma pigmentosum has a high risk of skin cancer, Cockayne syndrome presents with cachectic dwarfism and neurodegeneration, TTD shows brittle hair, susceptibility to infections, and no cancer risk, and COFS involves profound microcephaly with early lethality. Genetic testing targeting specific DNA repair pathway mutations confirms the diagnosis. To provide a broader perspective, it is important to acknowledge other rare syndromes, such as Menkes, Werner, and Netherton syndrome, which, while arising from different genetic etiologies, share some overlapping features such as neurodegeneration, premature aging, or skin abnormalities. Menkes disease, unlike TTD, is a disease of impaired copper transport, and clinical manifestations result from dysfunction in several copper‐dependent enzymes (Menkes 1988). Netherton Syndrome is a rare, autosomal recessive skin condition marked by congenital erythroderma, a distinct hair shaft anomaly, and atopic symptoms with elevated IgE levels (Bitoun et al. 2002). Werner Syndrome patients not only display signs of rapid aging (such as early graying and thinning of hair, skin atrophy) but also suffer from several age‐related disorders, including bilateral cataracts, diabetes mellitus, osteoporosis, early arteriosclerosis, and a range of benign and malignant tumors (Oshima et al. 1996). These similarities underscore the complexity of understanding genotype‐phenotype correlations in these disorders and highlight the need for further comparative studies to delineate shared and divergent pathways that contribute to their pathophysiology.

## Author Contributions

Data collection: Ali Reza Tavasoli, Arastoo Kaki, and Kara S. Lewis. Clinical evaluation: Ali Reza Tavasoli, Kara S. Lewis, Morteza Heidari, Mahmoud Reza Ashrafi and Arastoo Kaki. Genetic evaluation: Arastoo Kaki, Maedeh Ganji, Pouria Mohammadi, and Seyyed Mohammad Kahani. Conceptualization: all authors. Data analyses: Ali Reza Tavasoli, Arastoo Kaki, Maedeh Ganji, Seyyed Mohammad Kahani, Foozhan Radmehr, Pouria Mohammadi, and Kara S. Lewis. Conducting the experiments: Ali Reza Tavasoli, Arastoo Kaki, Maedeh Ganji, and Kara S. Lewis. Manuscript preparation: all authors. Approved the final manuscript. Ali Reza Tavasoli, Arastoo Kaki, Maedeh Ganji, Pouria Mohammadi, and Kara S. Lewis.

## Ethics Statement

The written informed consent was received from each guardian and they also provided a signed written consent to publish all personal and medical details included in this study.

## Conflicts of Interest

The authors declare no conflicts of interest.

## Data Availability

We state that the data not provided in this article will be shared on request by email to the corresponding author from any qualified investigator for purposes of replicating procedures and results.

## References

[mgg370067-bib-0001] Bitoun, E. , S. Chavanas , A. D. Irvine , et al. 2002. “Netherton Syndrome: Disease Expression and Spectrum of SPINK5 Mutations in 21 Families.” Journal of Investigative Dermatology 118, no. 2: 352–361. 11841556 10.1046/j.1523-1747.2002.01603.x

[mgg370067-bib-0002] Botta, E. , T. Nardo , D. Orioli , et al. 2009. “Genotype–Phenotype Relationships in Trichothiodystrophy Patients With Novel Splicing Mutations in the XPD Gene.” Human Mutation 30, no. 3: 438–445. 10.1002/humu.20912. 19085937

[mgg370067-bib-0003] Boyle, J. , T. Ueda , K. S. Oh , et al. 2008. “Persistence of Repair Proteins at Unrepaired DNA Damage Distinguishes Diseases With ERCC2 (XPD) Mutations: Cancer‐Prone Xeroderma Pigmentosum vs. Non‐Cancer‐Prone Trichothiodystrophy.” Human Mutation 29, no. 10: 1194–1208. 18470933 10.1002/humu.20768 PMC3477783

[mgg370067-bib-0004] Broughton, B. C. , H. Steingrimsdottir , C. A. Weber , and A. R. Lehmann . 1994. “Mutations in the Xeroderma Pigmentosum Group D DNA Repair/Transcription Gene in Patients With Trichothiodystrophy.” Nature Genetics 7, no. 2: 189–194. 10.1038/ng0694-189. 7920640

[mgg370067-bib-0005] Chatterjee, N. , and G. C. Walker . 2017. “Mechanisms of DNA Damage, Repair, and Mutagenesis.” Environmental and Molecular Mutagenesis 58, no. 5: 235–263. 28485537 10.1002/em.22087 PMC5474181

[mgg370067-bib-0006] De Boer, J. , J. De Wit , H. Van Steeg , et al. 1998. “A Mouse Model for the Basal Transcription/DNA Repair Syndrome Trichothiodystrophy.” Molecular Cell 1, no. 7: 981–990. 10.1016/S1097-2765(00)80098-2. 9651581

[mgg370067-bib-0007] Faghri, S. , D. Tamura , K. H. Kraemer , and J. J. DiGiovanna . 2008. “Trichothiodystrophy: A Systematic Review of 112 Published Cases Characterises a Wide Spectrum of Clinical Manifestations.” Journal of Medical Genetics 45, no. 10: 609–621. 10.1136/jmg.2008.058743. 18603627 PMC3459585

[mgg370067-bib-0008] Farmaki, E. , N. Nedelkopoulou , F. Delli , K. Sarafidis , and D. I. Zafeiriou . 2017. “Brittle Hair, Photosensitivity, Brain Hypomyelination and Immunodeficiency: Clues to Trichothiodystrophy.” Journal of Theoretical Biology 84: 89–90. 10.1007/s12098-016-2209-9 27473476

[mgg370067-bib-0009] Harreld, J. , E. Smith , N. Prose , P. Puri , and D. J. Barboriak . 2010. “Trichothiodystrophy With Dysmyelination and Central Osteosclerosis.” American Journal of Neuroradiology 31, no. 1: 129–130. 10.3174/ajnr.A1665. 20075106 PMC7964056

[mgg370067-bib-0010] Hashimoto, S. , and J. M. Egly . 2009. “Trichothiodystrophy View From the Molecular Basis of DNA Repair/Transcription Factor TFIIH.” Human Molecular Genetics 18, no. R2: R224–R230. 19808800 10.1093/hmg/ddp390

[mgg370067-bib-0011] Itin, P. H. , A. Sarasin , and M. R. Pittelkow . 2001. “Trichothiodystrophy: Update on the Sulfur‐Deficient Brittle Hair Syndromes.” Journal of the American Academy of Dermatology 44, no. 6: 891–924. 10.1067/mjd.2001.114294. 11369901

[mgg370067-bib-0012] Ji, H. , D. Li , Y. Wu , et al. 2018. “Hypomyelinating Disorders in China: The Clinical and Genetic Heterogeneity in 119 Patients.” PLoS One 13, no. 2: e0188869. 29451896 10.1371/journal.pone.0188869 PMC5815574

[mgg370067-bib-0013] Kleijer, W. J. , V. Laugel , M. Berneburg , et al. 2008. “Incidence of DNA Repair Deficiency Disorders in Western Europe: Xeroderma Pigmentosum, Cockayne Syndrome and Trichothiodystrophy.” DNA Repair 7, no. 5: 744–750. 10.1016/j.dnarep.2008.01.014. 18329345

[mgg370067-bib-0014] Lambert, W. C. , C. E. Gagna , and M. W. Lambert . 2010. “Trichothiodystrophy: Photosensitive, TTD‐P, TTD, Tay Syndrome.” Advances in Experimental Medicine and Biology 658: 106–110. 10.1007/978-1-4419-6448-9_10 20687499

[mgg370067-bib-0015] Lehmann, A. R. , C. F. Arlett , B. C. Broughton , et al. 1988. “Trichothiodystrophy, a Human DNA Repair Disorder With Heterogeneity in the Cellular Response to Ultraviolet Light.” Cancer Research 48, no. 21: 6090–6096. 2458832

[mgg370067-bib-0016] Liang, C. , K. H. Kraemer , A. Morris , et al. 2005. “Characterization of Tiger Tail Banding and Hair Shaft Abnormalities in Trichothiodystrophy.” 52, no. 2: 224–232. 10.1016/j.jaad.2004.09.013 15692466

[mgg370067-bib-0017] Stefanini, M. , E. Botta , M. Lanzafame , and D. Orioli . 2010. “Trichothiodystrophy: from basic mechanisms to clinical implications.” DNA Repair 9, no. 1: 2–10. 10.1016/j.dnarep.2009.10.005.. 19931493

[mgg370067-bib-0018] Maeda, N. 2015. “Proteoglycans and Neuronal Migration in the Cerebral Cortex During Development and Disease.” Frontiers in Neuroscience 9: 00098. 10.3389/fnins.2015.00098. PMC4369650 25852466

[mgg370067-bib-0019] Menkes, J. H. 1988. “Kinky Hair Disease: Twenty Five Years Later.” Development 10, no. 2: 77–79. 10.1016/s0387-7604(88)80074-3 2839049

[mgg370067-bib-0020] Meyer‐Puttlitz, B. , E. Junker , R. U. Margolis , and R. K. Margolis . 1996. “Chondroitin Sulfate Proteoglycans in the Developing Central Nervous System. II. Immunocytochemical Localization of Neurocan and Phosphacan.” Journal of Comparative Neurology 366, no. 1: 44–54. 8866845 10.1002/(SICI)1096-9861(19960226)366:1<44::AID-CNE4>3.0.CO;2-K

[mgg370067-bib-0021] Morice‐Picard, F. , M. Cario‐André , H. Rezvani , D. Lacombe , A. Sarasin , and A. Taïeb . 2009. “New Clinico‐Genetic Classification of Trichothiodystrophy.” American Journal of Medical Genetics. Part A 149, no. 9: 2020–2030. 10.1002/ajmg.a.32902 19681155

[mgg370067-bib-0022] Nakano, E. , R. Ono , T. Masaki , et al. 2014. “Differences in Clinical Phenotype Among Patients With XP Complementation Group D: 3D Structure and ATP‐Docking of XPD In Silico.” Journal of Investigative Dermatology 134, no. 6: 1775–1778. 10.1038/jid.2014.14. 24418926

[mgg370067-bib-0023] Oshima, J. , C.‐E. Yu , C. Piussan , et al. 1996. “Homozygous and Compound Heterozygous Mutations at the Werner Syndrome Locus.” Human Molecular Genetics 5, no. 12: 1909–1913. 8968742 10.1093/hmg/5.12.1909

[mgg370067-bib-0024] Porto, L. , R. Weis , C. Schulz , P. Reichel , H. Lanfermann , and F. Zanella . 2000. “Tay's Syndrome: MRI.” Neuroradiology 42: 849–851. 11151695 10.1007/s002340000443

[mgg370067-bib-0025] Tolmie, J. , D. De Berker , R. Dawber , et al. 1994. “Syndromes Associate With Trichotodystrophy.” Clinical Dysmorphology 3, no. 1: 1–14. 10.1097/00019605-199401000-00001. 8205320

[mgg370067-bib-0026] Usuda, T. , M. Saijo , K. Tanaka , N. Sato , M. Uchiyama , and T. Kobayashi . 2011. “A Japanese Trichothiodystrophy Patient With XPD Mutations.” Journal of Human Genetics 56, no. 1: 77–79. 20944642 10.1038/jhg.2010.123

